# Caffeine improves the shooting performance and reaction time of first-person shooter esports players: a dose-response study

**DOI:** 10.3389/fspor.2024.1437700

**Published:** 2024-07-10

**Authors:** Ethan J. Rogers, Michael G. Trotter, Daniel Johnson, Ben Desbrow, Neil King

**Affiliations:** ^1^ School of Exercise and Nutrition Sciences, Queensland University of Technology, Brisbane, QLD, Australia; ^2^ Department of Psychology, Umeå University, Umeå, Sweden; ^3^ School of Computer Science, Queensland University of Technology, Brisbane, QLD, Australia; ^4^ School of Health Sciences and Social Work, Griffith Health, Griffith University, Gold Coast, QLD, Australia; ^5^ Menzies Health Institute Queensland, Griffith University, Gold Coast, QLD, Australia

**Keywords:** esports performance, esports, caffeine, first-person shooter (FPS), video game, reaction time

## Abstract

Caffeine is recognized as one of the most effective dietary ergogenic aids in sports, yet its evidence-based effectiveness in esports is unclear. This study investigated the effects of two different doses of caffeine on the shooting performance and reaction time of 24 first-person shooter (FPS) esports players (22 men, 2 women; age = 22.29 ± 2.91 years). Participants completed three experimental trials in which they consumed either a water control (CON), a 1 mg·kg^−1^ BM (CAF1) or a 3 mg·kg^−1^ BM (CAF3) dose of caffeine. Performance measures (e.g., score, accuracy (%), hit rate (hits/sec), and shots fired) were assessed in a static clicking and reactive tracking style task on the KovaaK's FPS aim trainer. Reaction time was used to assess vigilance on the psychomotor vigilance test (PVT). Performance was measured at four time points in each trial: pre-treatment (PRE), 60 min (POST1), 80 min (POST2), and 100 min (POST3) post-treatment. Significant differences were identified using repeated-measures analysis of variances. Caffeine, irrespective of dose, significantly improved performance compared to CON for static clicking score and hit rate, reactive tracking accuracy, and reaction time on the PVT. Significant interactions between treatment and time were identified and *post hoc* analyses showed that compared to CON, CAF1 significantly improved static clicking score at POST1 and POST3, static clicking hit rate at POST1, reactive tracking accuracy at POST1, POST2, and POST3, and reaction time on the PVT at POST1 and POST2. *Post hoc* analysis also showed that compared to CON, CAF3 significantly improved static clicking score, reactive tracking accuracy, and reaction time on the PVT at all time points, in addition to static clicking hit rate at POST1 and POST3. In summary, caffeine supplementation enhances the shooting performance and reaction time of FPS esports players.

## 1 Introduction

Esports can be defined as “organized competitive digital gaming, played on a spectrum of professionalism” (1). To date, the performance enhancement strategies employed within esports are typically adapted from those of traditional sports, although the extent of their transferability is yet to be elucidated. Notably, caffeine is recognized as one of the most effective dietary ergogenic aids in sports (2–6), yet its evidence-based effectiveness in esports is unclear.

Caffeine (1,3,7-trimethylxanthine) is one of the most widely consumed substances globally (7, 8). Primarily consumed as coffee, caffeine is also found in a variety of products including tea, energy drinks, soft drinks, supplements, and sports foods (7). The caffeine content in these products typically ranges from 5 to 500 mg (8–10). Caffeine is a psychoactive substance with its stimulatory effects predominantly attributed to adenosine receptor inhibition (7, 11). Adenosine, an important neuromodulator in the brain, acts as a potent inhibitor of excitatory neurotransmitters (12, 13). Following ingestion, peak caffeine plasma concentrations are typically reached within 60 min, although individual variability has been shown to range between 15 and 120 min (14). Caffeine has a half-life of normally 3 to 5 h, exerting its effects for prolonged periods of time (11).

Caffeine's impact on performance can be partially explained by the empirical relationship between arousal and performance, depicted by the classic inverted U-shaped curve, where both low and high levels of arousal are associated with decreased performance (15, 16). Consequently, the efficacy of caffeine may exhibit dose-dependent characteristics and may also be influenced by an individual's baseline level of arousal (16). Enhanced physical performance is typically observed following caffeine doses ranging between 3 and 6 mg·kg^−1^ [Body Mass (BM)], while cognitive performance sees improvement at lower doses typically ranging between 0.5 and 4 mg·kg^−1^ BM, with doses exceeding these values potentially having adverse effects (11). Common negative side effects of caffeine, such as anxiety and gastrointestinal distress, are usually attributed to high doses or increased sensitivity in non-regular caffeine consumers (11, 17).

Caffeine's effect on cognition has been extensively reviewed, demonstrating cognitive performance enhancement in sports (18) and military personnel (19), reducing the risk of cognitive decline and dementia (20), and counteracting the effects of sleep deprivation (21). A general consensus is that caffeine, in doses ranging from 0.5 to 4 mg·kg^−1^ BM, improves fundamental aspects of cognitive functions such as reaction time, attention, and vigilance (11). Esports players, particularly at the professional level, excel in their cognitive abilities which are considered important contributors to their success (22–25). Video game players and esports players regularly consume caffeine, particularly in the form of coffee and energy drinks (26–29), often to combat fatigue or tiredness, increase energy or arousal, or improve concentration or skill (29, 30).

There has been emerging interest in exploring the use of caffeine as an ergogenic aid for video game and esports players (31–35). In two of these studies, researchers showed potential for the use of caffeine to improve performance, primarily via the use of traditional measures of cognitive performance, although the findings are equivocal (34, 35). The sensitivity of traditional cognitive tests to detect changes in esports players has been questioned, and it is suggested that game-based tests may be more appropriate (36). Recently, FPS aim trainers have emerged as a tool to assess shooting performance in video game and esports players (37). FPS aim trainers are designed to improve players' mechanical aiming skills in FPS video games and are often used by professional FPS esports players. FPS aim trainers offer tasks that simulate various shooting scenarios in a controlled environment, often with customizable settings such as target size, speed, and movement patterns. Various FPS aim trainers, such as internal firing ranges within FPS games like CS:GO, or external applications like KovaaK's and Aimlabs, are available.

To our knowledge, three studies have utilized FPS aim trainers to investigate the effects of caffeine on the shooting performance of video game and esports players (31–33). The first study, involving 15 professional esports players (7 Fortnite players and 8 CS:GO players), found that an acute 3 mg·kg^−1^ BM dose of caffeine significantly enhanced hit time and hit accuracy on a shooting task, and simple reaction time in a cognitive test (33). The second study, which involved nine recreational gamers, showed that an acute 125 mg dose of caffeine (∼1.5 mg·kg^−1^ BM) significantly improved the time to eliminate targets in a visual capacity threshold shooting task and time on target in a tracking task when compared to a placebo (32). However, this study (32) found that most measures of shooting performance were not influenced by caffeine. The findings of the most recent study are less conclusive, as results showed that an acute 200 mg dose of caffeine improved the number of targets hit in one shooting task only, when compared to a placebo, while other measures saw significant improvements pre- to post-dose in both the caffeine and placebo conditions (31).

Although these studies suggest caffeine might improve shooting performance in FPS video game and esports players, the benefits remain unclear. Additionally, no studies have manipulated caffeine doses to evaluate the efficacy of lower doses. Since cognitive enhancement is demonstrated at low to moderate caffeine doses (e.g., 0.5–4.0 mg·kg^−1^ BM) (11), performance benefits at lower doses may reduce the motivation for higher caffeine intake. Therefore, using a within-subject design to account for inter-individual variation, the primary aim of this study was to investigate the effects of two different acute doses of caffeine (1 mg·kg^−1^ BM and 3 mg·kg^−1^ BM), compared to a water control, on measures of shooting performance in FPS esports players. Additionally, as a comparable and traditional measure of cognitive performance, we aimed to assess the effects of caffeine on reaction time as a measure of vigilance using a PVT.

## 2 Methods

### 2.1 Study design

A within-subject repeated measures, randomized, counterbalanced, controlled, single-blinded study was conducted using 24 FPS esports players. Participants completed three experimental trials involving the oral administration of either a water control (CON), a 1 mg·kg^−1^ BM (CAF1), or a 3 mg·kg^−1^ BM (CAF3) dose of anhydrous caffeine administered via a capsule. The chosen doses fall within the 0.5 to 4 mg·kg^−1^ BM range, which is known to improve cognitive performance (11). A 3 mg·kg^−1^ BM caffeine dose has previously been shown to enhance shooting performance in similar tasks (33). The 1 mg·kg^−1^ BM dose was selected as a more realistic lower dose for this population. The experimental trials were separated by between two to four days to allow participants to return to their habitual caffeine intakes and preceded by three familiarization sessions separated by 24 h, with the first experimental trial day scheduled two to four days following completion of the familiarization sessions.

The study was reviewed and approved by the University Human Research Ethics Committee, in compliance with the National Statement on Ethical Conduct in Human Research (approval No. 7187). The participants provided their written informed consent to participate in this study.

### 2.2 Participants

Inclusion criteria for this study were ≥18 years of age, ≥3 months experience playing FPS games using mouse and keyboard controls, currently playing ranked mode in an FPS game, and habitual low to moderate caffeine consumers (40–400 mg·day^−1^ from food and beverages). For the purposes of this study, individuals of any level of professionalism who play ranked mode in an FPS game were considered esports players. This criterion aligns with our adopted definition of esports as mentioned previously (1). Ranked game modes are competitive matches where players are matched based on skill level and performance is tracked through a ranking system (e.g., ladder), while non-ranked game modes are more casual. Individuals were excluded from participation in this study if they were a current smoker; pregnant or breastfeeding; had a known allergy, sensitivity, or experience adverse reactions to consuming caffeine in doses up to and including 3 mg·kg^−1^ BM; or having a known medical condition, neurodivergence, or taking medication that might put them at a high risk of an adverse reaction to caffeine ingestion. Participants were recruited via convenience and snowball sampling, including posts on relevant social media, advertisements on the university research recruitment page, presentations to esports students, and the posting of flyers on university noticeboards. Potential participants completed an initial questionnaire to confirm eligibility. Twenty-four volunteer participants completed this study [22 men, 2 women; age = 22.3 ± 2.9 years; weight = 83.4 ± 19.8 kg; body mass index (BMI) = 26.0 ± 6.2 kg/m^2^].

#### 2.2.1 Sample size power calculation

The only previous study to investigate a 3 mg·kg^−1^ BM acute dose of caffeine on an FPS task demonstrated large improvements in hit accuracy (ES Cohens *d* = 1.0) and hit time (ES Cohens *d* = 0.6) (33). Using a two-sided *α* of 0.05, a power (1-*β*) of 0.80, and an effect size of 0.6, it was estimated that 24 participants would be required to detect a significant difference in this study.

### 2.3 Pre-Experimental procedures

All start times were scheduled at the participant's convenience, ≥2 h after their usual wakeup time, and standardized within participants. This approach accommodated varying personal schedules (e.g., sleep, work, university, etc.) without disrupting usual sleep habits and ensured consistent session timing. Participants were assigned to one of six possible treatment orders in a balanced and randomized method using the website Randomization.com (available online at http://www.randomization.com).

#### 2.3.1 Online screening

All potential participants completed an online screening form in the Research Electronic Data Capture (REDCap®) system. The form included items on demographics, and information relating to the inclusion/exclusion criteria. Eligibility was confirmed by the research team after reviewing responses to the online screening form and confirming habitual caffeine intake via a caffeine frequency calculator (Professor B. Desbrow, personal communication, May 13, 2022). Eligible participants completed a second brief questionnaire to collect additional demographic information and regular sleep habits for scheduling purposes.

#### 2.3.2 Familiarization procedure

Participants completed three online familiarization sessions separated by 24 h. Before each session, participants were instructed to comply with pre-trial standardization procedures, which included abstaining from alcohol for 24 h and caffeine from 6 pm the prior evening, refraining from vigorous exercise after waking up, and maintaining their usual bedtime. Participants received a free copy of KovaaK's via Steam (https://store.steampowered.com). In the first session, participants were instructed on completing the sessions and adjusting visual game settings (e.g., color theme) for consistency within and between participants. Mouse sensitivity and video settings (e.g., resolution) varied according to individual preferences and monitor differences. Participants then completed 20 attempts at each of the two shooting tasks, split into blocks of five, as previous research shows that shooting accuracy plateaus after a comparable period (38). Participants were allowed brief screen breaks between tasks if needed.

### 2.4 Experimental trial procedure

All experimental trials were conducted at the university's games research laboratory. Prior to each experimental trial, participants followed the same pre-trial standardization procedures as in the familiarization sessions, in addition to consuming ≥2 L of water in the 24 h prior to the experimental trial to reduce the likelihood of dehydration, which negatively impacts cognitive performance (39). For the initial experimental trial, participants were also instructed to keep a dietary record from the time they woke up until they arrived at the laboratory. Participants were instructed to replicate their dietary habits before the two subsequent experimental trials. No food or beverages, except water, were permitted during the experimental trials. Participants were instructed to bring their personal mouse and mousepad for comfort and performance optimization. A Logitech G305 Lightspeed Wireless Gaming Mouse and a Logitech G240 Cloth Gaming Mouse Pad were provided to participants who did not bring their own.

**Figure 1:**
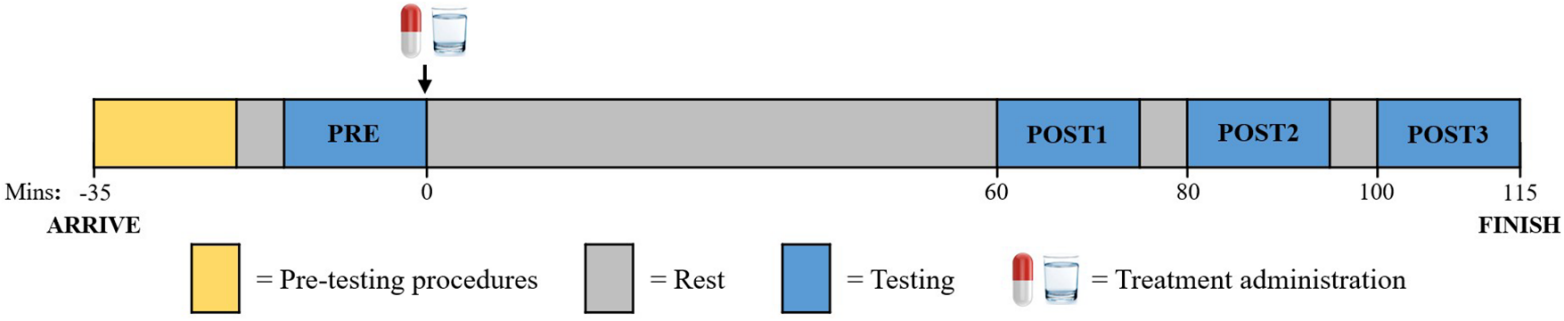
Experimental Trial Procedure.

The procedure for the experimental trials is shown schematically in Figure 1. Participants arrived at the laboratory at a scheduled convenient time. On arrival for the first experimental trial, participants confirmed compliance with pre-trial standardization procedures before their body weight was measured (used to calculate the caffeine dose). Subsequently, participants ingested 500 ml of water to further reduce the likelihood of dehydration. Participants then adjusted their mouse sensitivity and commenced a 10-min warmup on KovaaK's. The warm-up (five attempts at the two shooting tasks), was undertaken as previous research indicated rapid improvement in shooting occurred within the first few attempts each day (38). Following the warm-up, participants rested for five minutes before commencing baseline testing (PRE). Upon completion of PRE, participants ingested one of the assigned treatments and rested for 60 min to allow time for caffeine absorption. Participants then completed three more rounds of testing (POST1, POST2, POST3), separated by 5-min rest periods. Three rounds of testing were included post-treatment ingestion to understand how caffeine influences performance over a prolonged period. During the rest periods, participants were not permitted to engage in any mentally fatiguing activities (e.g., playing games), and were allowed to watch videos or browse social media. Each round of testing was identical, lasting 15 min, and included three tasks: two shooting tasks on KovaaK's (Static Clicking and Reactive Tracking), and a computerized PVT. Each task lasted five minutes, thus, the time each task began was staggered and is described relative to treatment ingestion (0 min) in Table 1. At the beginning of each round of testing, participants recorded their subjective ratings of alertness and tiredness.

**Table 1:** Timing of each task and round.

Task	Testing round			
	PRE	POST1	POST2	POST3
Static clicking	−15 min	60 min	80 min	100 min
Reactive tracking	−10 min	65 min	85 min	105 min
PVT	−5 min	70 min	90 min	110 min

All times are relative to treatment ingestion (0 min). PVT, psychomotor vigilance test.

### 2.5 Performance outcome measures

#### 2.5.1 KovaaK's shooting tasks

Shooting tasks in aim trainers are categorized into three domains: clicking, tracking, and target switching. Clicking involves eliminating targets quickly and accurately, either through single or multi-click actions. Subcategories of clicking include static clicking (stationary targets) and dynamic clicking (moving targets), with relevance to low time-to-kill (TTK) games like Counter-Strike and Valorant. Tracking requires players to keep their crosshair on the target while responding to target directional changes. Subcategories of tracking include smooth tracking (focused on smooth mouse control without unnecessary adjustments) and reactive tracking (quick reactions to sporadic target directional changes), with relevance to higher TTK games like Overwatch. Target switching combines clicking and tracking skills, demanding quick transitions between targets with varying TTKs. Subcategories of target switching include speed target switching (fast movements between targets) and evasive target switching (higher TTK targets that move more evasively), with relevance to lower TTK games where a higher number of enemies may be present at one time like Call of Duty and Apex Legends. An additional subcategory that can apply to all main categories is movement tasks, incorporating character movement such as strafing (sideways movements) which applies to all FPS games.

This study explored the effects of caffeine on static clicking and reactive tracking performance. Two shooting tasks were chosen from the “Online Scenarios” tab in KovaaK's, named “1wall6targets TE” and “1wall5targets pasu track invincible” which can be found by searching these terms via the search bar under the “Online Scenarios” tab on KovaaK's. Recently, KovaaK's has shown to be a reliable metrics platform for assessing shooting proficiency in FPS esports players (37). For the purposes of this study and simplicity, “1wall6targets TE” will be referred to as “Static Clicking”, and “1wall5targets pasu track invincible” will be referred to as “Reactive Tracking”.

##### 2.5.1.1 Static clicking

**Figure 2:**
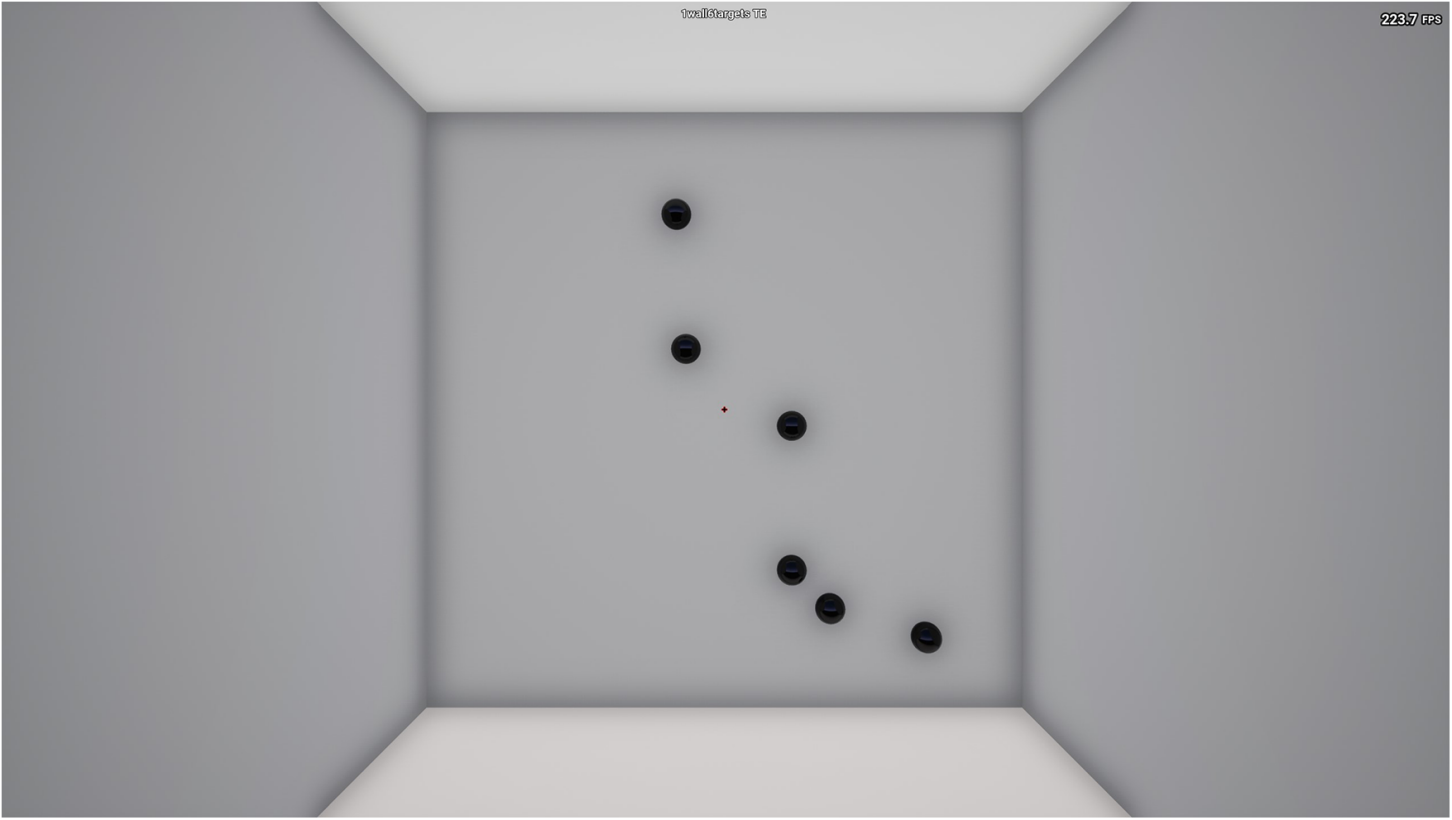
Static Clicking (1wall6targets TE) KovaaK's Shooting Task.

In this shooting task, the participant was presented with six targets on the screen that spawned in random locations within the field of view (FOV) (Figure 2). The objective was to eliminate the targets as quickly and accurately as possible for the entire 60-second duration. Participants eliminated targets by moving their crosshair over the target and clicking to shoot. Following a successful elimination, a new target spawned in a random location within FOV, hence there were six targets simultaneously present at any time. The targets had an unlimited duration and participants decided the sequence they eliminated the targets. Participants were instructed to achieve the highest score possible for this task, where the score equaled the number of eliminations (i.e., shots hit) multiplied by their accuracy (%). The rationale of this instruction was to avoid participants attempting to strategically prioritize either speed or accuracy. Performance outcome variables included for analysis were score [shots hit × accuracy (%)], accuracy (%) (shots hit ÷ shots fired × 100), hit rate (hits/sec) (shots hit ÷ 60 s), and shots fired.

##### 2.5.1.2 Reactive tracking

**Figure 3:**
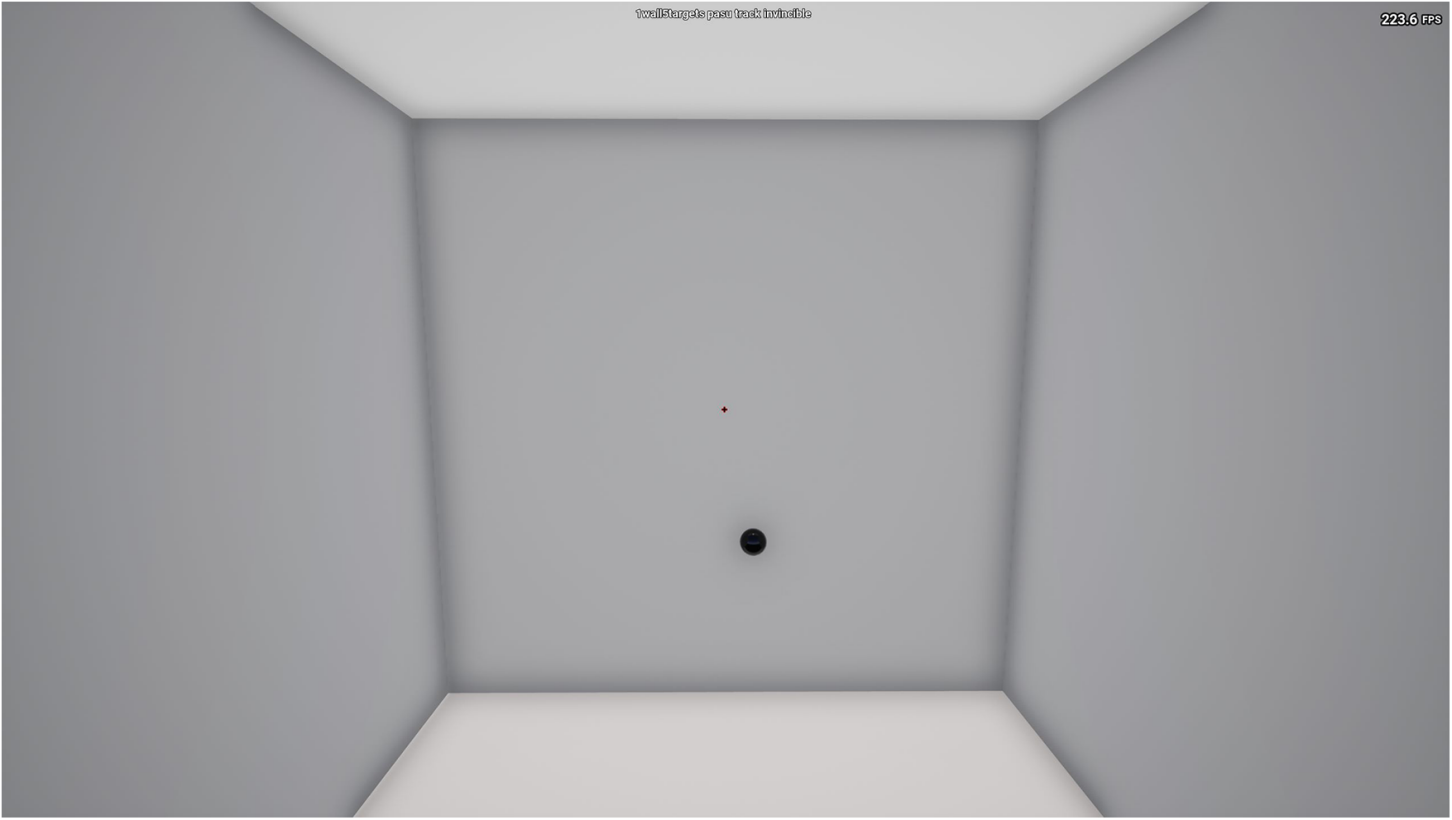
Reactive Tracking (1wall5targets pasu track invincible) KovaaK's Shooting Task.

In this shooting task, the participant was presented with one target on the screen that spawned in a random location within the FOV (Figure 3). The objective was for participants to maintain their crosshair placement on the target for the entire 60-second duration as it continuously moved around the screen with sporadic directional changes. Participants were instructed to click and hold the mouse button down for the entire duration for an accurate representation of their accuracy (i.e., time on target). The performance outcome variable was accuracy (%) (shots hit ÷ shots fired × 100).

#### 2.5.2 Psychomotor vigilance test (PVT)

The PVT is a frequently utilized assessment of vigilance that measures simple reaction time, lapses [reaction time > 500 milliseconds (ms)], and false starts to stimuli, that occur in random intervals for a prolonged period. The PVT was administered via a computer (available online at www.millisecond.com/download/library) and participants were instructed to respond to the stimuli as fast as possible by pressing the spacebar. The PVT shows no learning curve or influence by aptitude (40) and was therefore not included in the familiarization sessions. A 5-min PVT was used in this study as a suitable substitute for the usual 10-min test (41). The performance outcome variable for this task was average reaction time (ms).

### 2.6 Subjective ratings

Participants' subjective ratings of alertness and tiredness were obtained using 100 mm visual analog scales (VAS), where zero represented “not at all” and 100 represented “extremely”.

### 2.7 Treatments

The two caffeine treatments were: 1 mg·kg^−1^ BM anhydrous caffeine (PCCA, Australia) consumed with 250 ml water; and 3 mg·kg^−1^ BM anhydrous caffeine consumed with 250 ml water, both administered via a capsule. These doses equated to 83 ± 20 mg and 250 ± 60 mg respectively for the participants in this study. In the control treatment, participants consumed 250 ml of plain water (no capsule). A pure control was chosen over a placebo control for the ecological validity from a consumer perspective (i.e., consumers make a conscious decision to consume caffeine or not) and to act as an accurate control measure for comparative purposes of the two caffeine treatments.

### 2.8 Data management

For each participant, the average score from each round (i.e., PRE, POST1, POST2, and POST3) was computed for all performance outcome variables, and used in subsequent analyses (i.e., the average of five attempts), yielding 12 data points for each variable (one per round, with four rounds per trial, and three trials). This approach ensures a more accurate depiction of participants' true skill level while mitigating the known influence of sub-optimal effort on performance (42). Subjective ratings were analyzed by comparing the average scores of POST1, POST2, and POST3 between treatments.

### 2.9 Statistical analyses

Statistical analysis was performed using IBM SPSS Statistics (Version 29). Normality was tested on studentized residuals using the Shapiro-Wilk test. Outliers were identified by studentized residuals >±3. Two-way repeated measures analysis of variances (RM-ANOVAs) were performed to evaluate the effects of treatment (CON, CAF1, CAF3) and time (PRE, POST1, POST2, POST3) on each performance outcome variable. One-way RM-ANOVAs were performed to evaluate the effects of treatment on subjective ratings. Statistical significance was accepted as *p* < .05, and significant differences were identified with *post hoc* tests adjusted for multiple comparisons (Bonferroni). Sphericity was tested using Mauchly's test of sphericity and the degrees of freedom were corrected using the Greenhouse-Geisser method if not met. Data were log-transformed and re-analyzed if non-normally distributed (Shapiro-Wilk test, *p *< .05). If the log-transformation did not improve normality, non-parametric Friedman tests were used to evaluate the effects of treatment and time on performance, with statistical significance accepted as *p* < .05. Data are reported as means ± standard deviations (SD) when normally distributed and medians (and inter-quartile range, IQR) when non-normally distributed. Reliability analysis was performed using the intraclass correlation coefficient (ICC) on the three baseline measures (PRE) from each experimental trial. ICC estimates and their 95% confidence intervals (CI) were calculated based on a mean rating (*k* = 3), absolute agreement, two-way mixed-effects model. ICC values < 0.5 indicate poor reliability, between 0.5 and 0.75 indicate moderate reliability, between 0.75 and 0.9 indicate good reliability, and values > 0.9 indicate excellent reliability (43).

## 3 Results

### 3.1 Participant characteristics

Twenty-four participants (22 male, 2 female; age = 22.3 ± 2.9 years; weight = 83.4 ± 19.8 kg; BMI = 26.0 ± 6.2 kg/m^2^) completed the study. Participants' self-reported habitual caffeine intake was 209 ± 102 mg/day. Participants played ranked game modes across Valorant (*n* = 8), CS:GO (*n* = 7), Overwatch (*n* = 7), Counter-Strike 2 (*n* = 5), Apex Legends (*n* = 2), Halo Infinite (*n* = 2), and Paladins (*n* = 1), with some participants playing ranked game modes in multiple FPS games. The rank distributions of participants within the respective FPS esports titles are detailed in Appendix A.

### 3.2 Reliability testing

All performance outcome variables showed excellent reliability (95% CIs > 0.90), except for accuracy on the Static Clicking task which showed good to excellent reliability (95% CI 0.76–0.94).

### 3.3 Effects of caffeine on shooting performance

All assumptions of RM-ANOVAs were met unless otherwise specified. There were no significant differences at baseline (PRE) between treatments for all performance outcome variables (*p* ≥ .534).

#### 3.3.1 Static clicking—score

**Figure 4:**
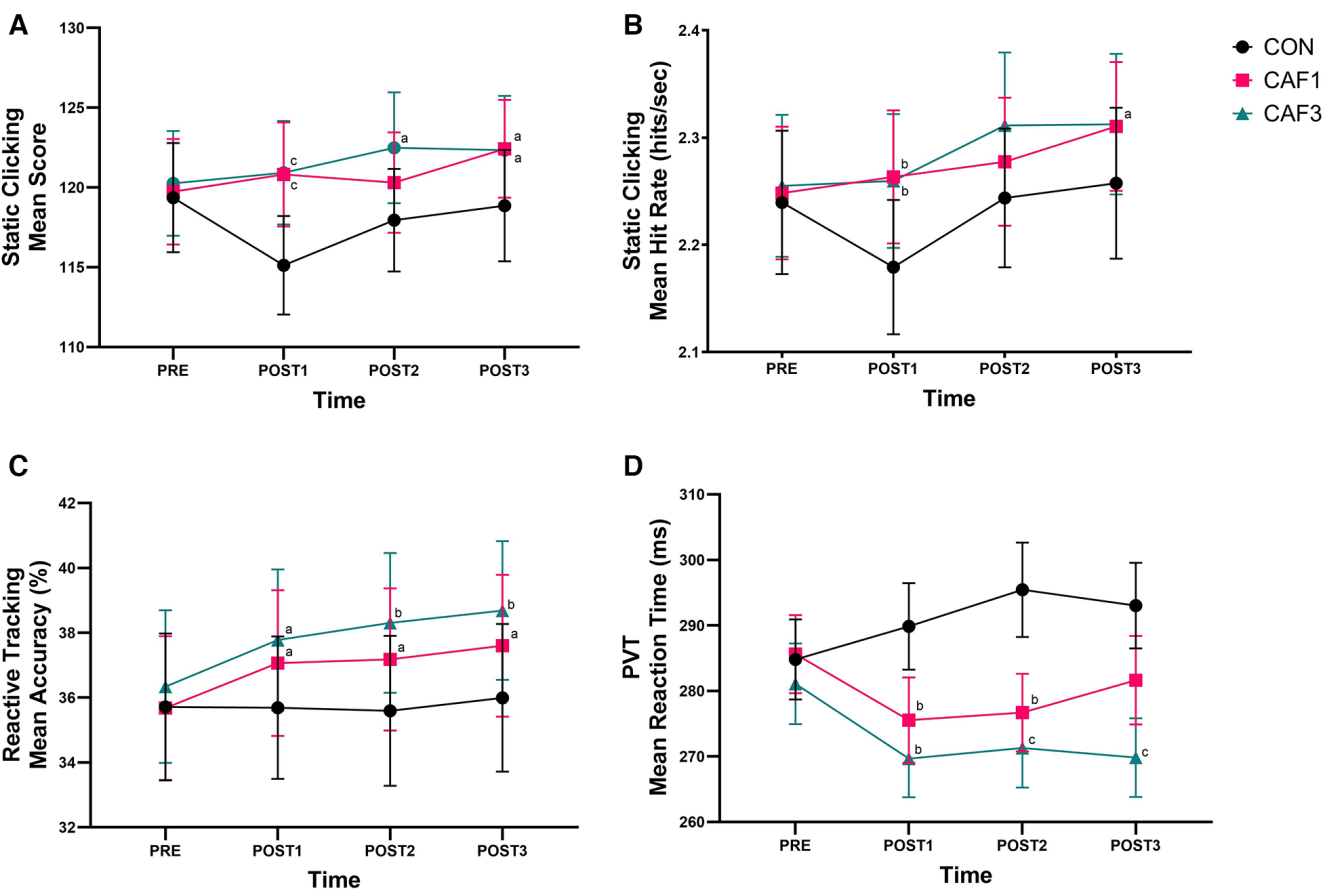
(**A**) Static clicking mean scores across time for each treatment. (**B**) Static clicking mean hit rate (hits/sec) across time for each treatment. (**C**) Reactive tracking mean accuracy (%) across time for each treatment. (**D**) PVT mean reaction time (ms) across time for each treatment. Score = shots hit ÷ accuracy (%); Hit rate (hits/sec) = shots hit ÷ 60 s; Accuracy (%) = shots hit ÷ shots fired × 100; Error Bars indicate SEM. CON: Control; CAF1: 1 mg per kg BM caffeine dose; CAF3: 3 mg per kg BM caffeine dose; ^a^ Significantly different to CON treatment at that time point (*p* < .05). ^b^ Significantly different to CON treatment at that time point (*p* < .01). ^c^ Significantly different to CON treatment at that time point (*p* < .001).

There was a significant main effect of treatment, *F*(2, 46) = 7.573, *p* = .001, partial *η*^2^ = .248. *Post hoc* analysis showed that score was significantly higher in CAF1 (120.8 ± 15.3, *p* = .023) and CAF3 (121.5 ± 16.0, *p* = .005) compared to CON (117.8 ± 15.8). There was a significant interaction between treatment and time, *F*(6, 138) = 2.543, *p* = .023, partial *η*^2^ = .100. *Post hoc* analysis showed that score was significantly higher in CAF1 (*p* < .001) and CAF3 (*p* < .001) compared to CON at POST1, in CAF3 (*p* = .044) compared to CON at POST2, and in CAF1 (*p* = .039) and CAF3 (*p* = .030) compared to CON at POST3 (Figure 4A).

#### 3.3.2 Static clicking—accuracy (%)

A Friedman test was run to determine if there were differences in static clicking accuracy (%) between treatments and individual time points across treatments. There was no significant difference between treatments (CON = 88.3 (4.3); CAF1 = 88.9 (3.84); CAF3 = 88.8 (5.06)), *χ*^2^(2) = 3.000, *p* > .05. There was no significant difference between time points, *χ*^2^(11) = 17.795, *p* > .05.

#### 3.3.3 Static clicking—hit rate (hits/sec)

There was a significant main effect of treatment, *F*(2, 46) = 6.913, *p* = 0.002, partial *η*^2^ = 0.231. *Post hoc* analysis showed that hit rate was significantly higher in CAF1 (2.28 ± 0.29, *p* = .022) and CAF3 (2.29 ± 0.32, *p* = .011) compared to CON (2.23 ± 0.32). There was a significant interaction between treatment and time, *F*(6, 138) = 3.090, *p* = 0.007, partial *η*^2^ = .118. *Post hoc* analysis showed that hit rate was significantly higher in CAF1 (*p* = .001) and CAF3 (*p* = .003) compared to CON at POST1, and in CAF3 (*p* = .02) compared to CON at POST3 (Figure 4B).

#### 3.3.4 Static clicking—shots fired

There was no significant main effect of treatment (CON = 152.4 ± 25.5, CAF1 = 154.7 ± 22.8, CAF3 = 155.1 ± 24.9, *p* > .05), or interaction between treatment and time (*p* > .05).

#### 3.3.5 Reactive tracking—accuracy (%)

There was a significant main effect of treatment, *F*(2, 46) = 10.142, *p* < .001, partial *η*^2^ = 0.306. *Post hoc* analysis showed the accuracy was significantly higher in CAF1 (36.9 ± 10.8, *p* = .041) and CAF3 (37.8 ± 10.7, *p* < .001) compared to CON (35.7 ± 11.0). Mauchly's test of sphericity was significant for the interaction between treatment and time, *χ*^2^(20) = 39.176, *p* = .007. There was a significant interaction between treatment and time, *F*(4.112, 94.575) = 2.583, *p* = .021, partial *η*^2^ = 0.101. *Post hoc* analysis showed that accuracy was significantly higher in CAF1 (*p* = .029) and CAF3 (*p* = .012) compared to CON at POST1, in CAF1 (*p* = .022) and CAF3 (*p* = .001) compared to CON at POST2, and in CAF1 (*p* = .023) and CAF3 (*p* = .002) compared to CON at POST3 (Figure 4C).

### 3.4 Effects of caffeine on psychomotor vigilance

#### 3.4.1 Reaction time

There was a significant main effect of treatment, *F*(2, 46) = 16.652, *p* < .001, partial *η*^2^ = .420. *Post hoc* analysis showed that reaction time was significantly faster in CAF1 (279.8 ± 29.1, *p* = .008) and CAF3 (273.0 ± 28.3, *p* < .001) compared to CON (290.8 ± 31.1). There was a significant interaction between treatment and time, *F*(6, 138) = 5.673, *p* < .001, partial *η*^2^ = .198. *Post hoc* analysis showed that reaction time was significantly faster in CAF1 (*p* < .001) and CAF3 (*p* = .002) compared to CON at POST1, in CAF1 (*p* = .001) and CAF3 (*p* < .001) compared to CON at POST2, and in CAF3 (*p* < .001) compared to CON at POST3 (Figure 4D).

### 3.5 Subjective ratings

There was no significant difference at baseline between treatments for alertness and tiredness. Mauchly's test of sphericity was significant for subjective ratings of alertness between treatments, *χ*^2^(2) = 7.936, *p* = .019. There was a significant difference in alertness between treatments, *F*(1.535, 35.308) = 31.870, *p* < .001, partial *η*^2^ = .581. *Post hoc* analysis showed that alertness was significantly higher in CAF1 (*p *< .001) and CAF3 (*p *< .001) compared to CON. There was no significant difference between caffeine treatments for alertness. There was a significant difference in tiredness between treatments, *F*(2, 46) = 18.320, *p* < .001, partial *η*^2^ = .443. *Post hoc* analysis showed that tiredness was significantly lower in CAF1 (*p *< .001) and CAF3 (*p *< .001) compared to CON. There was no significant difference between caffeine treatments for tiredness.

## 4 Discussion

To our knowledge, this study is the first to explore the effects of manipulating caffeine dose on the shooting performance of FPS esports players. The primary aim was to assess the effects of an acute 1 mg·kg^−1^ BM and 3 mg·kg^−1^ BM dose of caffeine on various outcome measures of shooting performance. Our overall finding suggests that irrespective of dose, acute caffeine supplementation improves shooting performance in FPS esports players. Specifically, both doses of caffeine improved performance in static clicking and reactive tracking shooting tasks. Caffeine also significantly improved reaction time in the PVT, in addition to self-reported ratings of alertness and tiredness, irrespective of dose.

While these results suggest that caffeine enhances the shooting performance of FPS esports players, they should be interpreted with caution, considering the study's methodological context and its differences from actual FPS esports gameplay. For instance, while static clicking is relevant to FPS titles such as Counter-Strike, and reactive tracking is relevant to FPS titles such as Overwatch, we acknowledge the nature of shooting tasks in KovaaK's differs significantly from the shooting requirements in FPS esports titles. Therefore, the results will be discussed within the confines of the methodology used.

The overall improvement in static clicking following caffeine administration was due to an increase in hit rate while maintaining a high level of accuracy. This finding aligns with previous research demonstrating improved hit time (average time needed to hit a target) following an acute 3 mg·kg^−1^ BM dose of caffeine in a similar shooting task (33). Such improvements may be attributed to an increase in motor speed that is typically seen following caffeine administration (44). However, contradictory findings have reported no significant differences in hit rate (hits/sec) with a 200 mg dose of caffeine compared to a placebo (31), and no significant difference in time to eliminate targets with a 150 mg dose of caffeine compared to placebo (32). The disparity may be explained by an intentional speed-accuracy trade-off, indicated by a consistent increase in the number of shots fired after caffeine ingestion (31). Notably, we did not find any significant differences in the number of shots fired between treatments, potentially due to methodological differences. For instance, in our study, participants were instructed to aim for the highest score possible, discouraging them from prioritizing either speed or accuracy exclusively. Another disparity was that our study did not find any statistically significant differences in static clicking accuracy between treatments, contrary to previous reports of significant improvements in accuracy following a 3 mg·kg^−1^ BM dose of caffeine, compared to placebo (33). One potential explanation could be the difference in the shooting task employed previously (33), which had a finite number of targets (60 targets) compared to the finite task duration (60 s) in our study, making their test more sensitive to changes in accuracy. Interestingly, the previous authors (33) noted a potential ceiling effect with both placebo and caffeine treatments reaching close to 100% accuracy, yet a significant difference was still observed with a large effect size.

Our findings also demonstrated that caffeine, irrespective of dose, improved reactive tracking via an increase in accuracy (i.e., time on target). This finding aligns with previous research where a 125 mg dose of caffeine improved time on target in a similar tracking task (32). To our knowledge, the cognitive demands associated with reactive tracking in FPS games have not been explored. However, we speculate that several cognitive functions and processes are involved in the effective execution of this task, such as vigilance, attention, and reaction time, which are known to benefit from caffeine administration (11). Given the necessity to quickly respond to the sporadic directional changes of the target in this task, it is not surprising that both reaction time and reactive tracking showed concurrent improvement in both caffeine treatments within the present study.

Similar to previous research (45), caffeine supplementation improved PVT performance, characterized by faster reaction times observed in both caffeine treatments compared to CON, indicating enhanced vigilance. This finding is unsurprising as most of the evidence supports the beneficial effects of caffeine on reaction time and vigilance (for review see; McLellan et al., 2016). In essence, reaction time is a measure of the duration between a stimulus and the execution of a motor plan in response to that stimulus, integrating both neurological and motor processes. It has been suggested that caffeine's effect on reaction time is driven by the acceleration of attentional rather than motor processes (46). Thus, the effects of caffeine on reaction time and potentially reactive tracking within our study may be predominantly influenced by improvements in neurological processes.

Notably, no significant differences were observed between caffeine doses across all performance measures in our study. This finding indicates that similar enhancements in shooting performance can be achieved with both a 1 mg·kg^−1^ BM and 3 mg·kg^−1^ BM dose of caffeine. This has practical implications for individuals who may be vulnerable to potential adverse side effects associated with caffeine consumption, such as increased anxiety and gastrointestinal disturbance, although typically reported at higher doses or in non-habitual caffeine consumers (11). However, it is worth noting that a marginal, albeit non-significant, improvement was observed with the 3 mg·kg^−1^ BM dose of caffeine compared to the 1 mg·kg^−1^ BM dose for reactive tracking accuracy (see Figure 4C) and reaction time in the PVT (see Figure 4D). These observations suggest a trend associated with greater improvements following a higher dose. This trend could have real-world implications where the performance differences between professional esports players and teams are negligible, meaning that even minimal improvements could provide a competitive edge. Thus, individuals may consider opting for a 3 mg·kg^−1^ BM dose over a 1 mg·kg^−1^ BM dose to potentially enhance their performance further. Individual consideration should also be given to the potential side effects of caffeine, although doses up to ∼4 mg·kg^−1^ BM generally improve cognition with minimal side effects (11).

Our findings also show that caffeine consistently improved performance 60–75 min post-ingestion (POST1), irrespective of dose, compared to CON. Significant enhancements were observed in static clicking score and hit rate, reactive tracking accuracy, and reaction time on the PVT. Collectively, this suggests that caffeine is most effective at 60–75 min post-ingestion. The results also imply that the 3 mg·kg^−1^ BM dose of caffeine may produce a more sustained and consistent effect on performance compared to the 1 mg·kg^−1^ BM dose. Notably, significant improvements were observed from the 3 mg·kg^−1^ BM dose at all time points for static clicking score, reactive tracking accuracy, and reaction time on the PVT, in comparison to CON. However, as mentioned previously, it is important to acknowledge that no statistically significant differences were found between the two caffeine doses at any time point. While there were other significant differences observed at individual time points between the caffeine treatments and CON, their inconsistency makes it challenging to draw definitive conclusions.

### 4.1 Limitations and direction for future research

The present investigation is subject to several limitations. Firstly, pre-trial standardization procedure checks relied on self-reports, including caffeine abstinence. Thus, we cannot be certain participants abstained from caffeine before attending. Previous research has shown that some participants may still have residual caffeine levels in their blood, despite acknowledging compliance with caffeine abstinence (45). Future studies may consider including plasma caffeine analysis to provide objective data on baseline plasma caffeine levels and to improve the intervention fidelity by showing whether the higher dose objectively increased the level of circulating caffeine compared to the lower dose. Secondly, our study specifically explored the effects of caffeine on the isolated shooting performance of FPS esports players, focusing on static clicking and reactive tracking shooting scenarios in KovaaK's. Therefore, the generalizability of our findings to actual FPS esports gameplay, or their applicability to other esports genres, remains uncertain. Furthermore, while our study implies that caffeine improves shooting performance in FPS esports players, it is reasonable to assume that not all individuals will experience these benefits, or at least not to the same extent. Further exploration into the individual variances in shooting performance following caffeine administration is needed. Additionally, the low number of female participants (*n* = 2) did not allow comparison between sexes. Previous research has also shown potential expectancy effects of caffeine (47), which were not measured within this study. Lastly, caffeine was administered in a non-conventional format (capsule), and we do not know if similar effects will be seen using more conventional sources of caffeine (e.g., coffee, energy drinks, etc.). As there is limited research within this space and some studies have shown strong industry involvement, there is a need for more independent research to expand our understanding of how caffeine and other strategies may influence esports performance.

## 5 Conclusion

In summary, our findings indicate that acute administration of both a 1 mg·kg^−1^ BM and 3 mg·kg^−1^ BM dose of caffeine improved performance across static clicking and reactive tracking shooting tasks, in addition to reaction time in a PVT. Results from our study also suggest that a 1 mg·kg^−1^ BM dose of caffeine produces a similar positive effect on shooting performance and reaction time compared to the 3 mg·kg^−1^ BM dose.

## Data Availability

The raw data supporting the conclusions of this article will be made available by the authors, without undue reservation.

## Appendix A

**Table A1:** Ranks of participants within their respective FPS esports titles.

FPS Esports title	Rank	Number of participants
Valorant	Ascendant	1
	Gold	3
	Silver	4
CS:GO	Legendary Eagle Master	2
	Gold Nova	1
	Silver	4
Overwatch	Top 500	1
	Diamond	1
	Platinum	3
	Gold	1
	Silver	1
Counter-Strike 2	Master Guardian	1
	Silver	4
Apex Legends	Diamond	1
	Gold	1
Halo Infinite	Onyx	2
Paladins	Gold	1
